# Detection of *Enterocytozoon bieneusi* in Non-Human Primates in Portuguese Zoos

**DOI:** 10.3390/ani14131874

**Published:** 2024-06-25

**Authors:** Guilherme Moreira, Andreia V. S. Cruz, Sérgio Santos-Silva, Rafaela S. S. Moreira, João R. Mesquita

**Affiliations:** 1 School of Medicine and Biomedical Sciences (ICBAS), University of Porto, 4050-313 Porto, Portugal; gmoreiravet@gmail.com (G.M.); up201303679@edu.icbas.up.pt (A.V.S.C.); up202110051@edu.icbas.up.pt (S.S.-S.); rafaelasimaomoreira@gmail.com (R.S.S.M.); 2 Epidemiology Research Unit (EPIUnit), Instituto de Saúde Pública da Universidade do Porto, 4050-600 Porto, Portugal; 3 Laboratory for Integrative and Translational Research in Population Health (ITR), 4050-600 Porto, Portugal

**Keywords:** *Enterocytozoon bieneusi*, microsporidia, zoo, non-human primates, Portugal

## Abstract

**Simple Summary:**

*Enterocytozoon bieneusi*, the leading cause of human microsporidiosis, was found in 1.57% of zoo animal samples from Portugal, exclusively in non-human primates, underscoring their potential contribution to the transmission of this microsporidia to humans and other animals.

**Abstract:**

*Enterocytozoon bieneusi*, an intracellular eukaryote closely related to fungi, is recognized as a significant pathogen affecting humans, particularly those with compromised immune systems. While its transmission routes are still not fully elucidated, fecal–oral transmission remains the primary one. With a wide host range, the zoonotic potential of *E. bieneusi* is a concern, albeit direct evidence of animal-to-human transmission remains scarce. Genotyping based on the internal transcribed spacer (ITS) region facilitates the delineation of genetic diversity, with potentially zoonotic genotypes predominantly associated with Groups 1 and 2. Despite the broad spectrum of susceptible animal hosts, research into microsporidian infection among zoo animals remains limited. This study aimed to evaluate the occurrence of *E. bieneusi* infection across diverse captive animals, focusing on zoo settings in Portugal. Fecal samples were collected from a variety of animals, and molecular detection of *E. bieneusi* was conducted using nested PCR targeting the ITS region. Of 127 fecal samples, 1.57% (95% CI: 0.19–5.57) tested positive for *E. bieneusi*, with non-human primates (NHP’s) exhibiting an 18.18% (95% CI: 2.28–51.78) occurrence. Phylogenetic analysis revealed clustering within Group 2 genotypes, indicating potential zoonotic implications. This study highlights the need for further research to understand the epidemiology of *E. bieneusi* in zoo environments and its potential transmission pathways to humans.

## 1. Introduction

Microsporidia are intracellular eukaryotic organisms closely related to fungi that demonstrate a wide host range spanning both invertebrates and vertebrates, with 220 genera described and more than 1700 species catalogued, 17 of which pose pathogenic risks to humans [1]. Of particular prominence in human infections, especially among immunocompromised individuals, is *Enterocytozoon bieneusi* [2]. Initial documentation of *E. bieneusi* as an intestinal pathogen in a HIV-infected patient dates back to 1985 [3], and subsequent studies highlighted its substantial impact on immunocompromised hosts, particularly those with AIDS or organ transplants, as it can lead to severe and life-threatening diarrhea and wasting syndrome [4,5,6,7].

Despite considerable research efforts, the precise modes of microsporidian transmission, including *E. bieneusi*, remain incompletely described [8,9,10,11,12,13]. Nonetheless, infection is acquired via fecal–oral transmission of spores either through direct contact or exposure to contaminated water or food [10]. As infection progresses, spores enter host enterocytes through the discharged polar tube, introducing the sporoplasm. This is followed by the development of meronts, and consequently multinucleated types. After that, these plasmodia undergo sporogony, producing sporoblasts. Fully formed spores emerge, leaving the affected cells and being eventually released through stool [8,14]. 

Zoonotic transmission is conceivable, given the demonstrated capacity of *E. bieneusi* to infect various domestic and wild animal hosts. However, direct evidence substantiating animal-to-human transmission remains elusive, notwithstanding documented infections across diverse animal species, including beavers, calves, cats, chickens, dogs, foxes, goats, llamas, macaques, muskrats, ostriches, otters, pigs, pigeons, rabbits, raccoons, and wild boars have been shown [8,15]. Furthermore, *E. bieneusi* infection in reptiles and amphibians is not well understood, although there are reports and molecular characterization of the microsporidian in captive snakes [16] and in edible bullfrogs (*Lithobates catesbeiana*) in China [17]. These findings underscore the potential reservoir role of these animals in the dissemination of microsporidian spores capable of infecting humans.

The conventional approach to *E. bieneusi* genotyping is based on the examination of polymorphisms within internal transcribed space (ITS) nucleotide sequences. This region is flanked by ribosomal RNAs, and exhibits notable variety across *E. bieneusi* isolates, facilitating the discernment of intraspecific genetic diversity [8]. Upwards of 600 genotypes of *E. bieneusi* have already been catalogued and stratified into 13 [18] phylogenetic groups. Potentially zoonotic genotypes tend to be associated with Groups 1 and 2, with Group 1 exhibiting the largest representation, comprising over 300 genotypes [10]. Recent investigations have demonstrated the presence of certain genotypes from Groups 1 and 2 across multiple host species, underscoring their broad zoonotic potential. Conversely, genotypes aligned with Groups 3 to 13 display a greater degree of host specificity, and consequently their impact on public health remains to be fully understood [19].

Despite the broad range of susceptible animal hosts, investigation into microsporidian infection among zoo animals remains limited in scope [20]. However, the easy access that both visitors and zookeepers have to zoo animals poses risks related to the transmission of zoonotic pathogens [20,21]. Therefore, the primary objective of this study is to evaluate the occurrence of *E. bieneusi* infection across a diverse array of captive animals, including birds, reptiles, amphibians, mammals, and arthropods. Additionally, the study aims to genetically characterize the circulating *E. bieneusi* genotypes in these zoo animals.

## 2. Materials and Methods

### 2.1. Sample Collection

This study screened 127 fecal samples from two Zoological Gardens (Maia Zoo and Pedagogical Farm of Canelas), where animals are housed for educational, recreational, and conservation objectives. Both are located in the Porto district of the northern region of mainland Portugal. Feces with a well-formed structure and no other signs of gastrointestinal disease were collected from the soil immediately after excretion by selectively extracting material from the inner core of the fecal matter. From the Maia Zoo, fecal samples (*n* = 76) were collected from 61 species of animals belonging to 39 different families (Supplementary Table S1). From the Pedagogical Farm of Canelas, fecal samples (*n* = 51) were collected from 12 species, from 10 families. Animals from both sites were exposed to regular proximity with human beings, often direct contact. Animals from the Pedagogical farm were subjected to frequent contact with visitors and caretakers. Animals from Maia Zoo had constrained yet recurrent interaction with human visitors, and frequent direct contact with caretaker staff. All samples, collected in September 2023, were immediately kept at −20 °C following collection until DNA extraction.

### 2.2. Nucleic Acid Extraction

Fecal suspensions (10%) were prepared in phosphate-buffered saline pH 7.2. The samples were then homogenized for 5 min using the Disruptor Genie (Scientific Industries, Inc., Bohemia, NY, USA) and then centrifuged for 5 min at 8000× *g*.

DNA extraction was carried out using 140 μL of the resultant supernatant and the QIAamp DNA Mini Kit (Qiagen, Hilden, Germany), according to the manufacturer’s instructions, in the automatic extraction machine QIAcube (Qiagen, Hilden, Germany). DNA was eluted in RNase-free water and kept at −20 °C until further analysis. Stools positive for *E. bieneusi* were extracted in each batch of 12 samples and used as PCR positive controls. 

### 2.3. Molecular Detection of Enterocytozoon bieneusi

Detection of *E. bieneusi* was performed using a nested PCR amplifying the internal transcribed spacer (ITS) region as well as the flanking small and large subunits of the ribosomal RNA (rRNA), with the outer primer set EBITS3/EBITS4 (435 bp) and the inner primer set EBITS1/EBITS2.4 (390 bp) (Table 1) [22].

### 2.4. General Procedures

All PCR reactions were run on T100 thermocycler (Bio-Rad, Hercules, CA, USA). Reaction mixtures were performed using the Speedy Supreme NZYTaq 2x Green Master Mix (NZYTech, Lisbon, Portugal), in accordance with the manufacturer’s instructions. The cycling conditions were as follows: initial denaturation at 95 °C for 3 min, 40 cycles of denaturation at 95 °C for 15 s, annealing at 57 °C for 15 s for the first round of PCR or 55 °C for 13 s for the second round, extension at 72 °C for 2 s and final extension at 72 °C for 10 min. The amplified DNA fragments were identified by electrophoresis on 1.5% agarose gels, stained with Xpert Green Safe DNA gel dye (GRiSP^®^, Porto, Portugal), at 100 V for 30 min. UV light irradiation was used to visualize the results.

### 2.5. Sequencing and Phylogenetic Analysis

Amplicons with the expected size were purified using GRS PCR & Gel Band Purification Kit (GRiSP^®^, Porto, Portugal). Following purification, bidirectional sequencing was carried out using the Sanger dideoxy sequencing method and the inner primers for the target gene. The obtained sequences were aligned using BioEdit Sequence Alignment Editor v7.2.3 software package and compared to those found in the NCBI nucleotide database (GenBank, retrieved on 6 February 2024). MEGA-X version 10.2.6 software [23] was used to calculate the pairwise distances between the sequences obtained in this study. MEGA-X version 10.2.6 software [23] and the interactive Tree of Life (iTOL) platform [24] were used for phylogenetic analysis, including representative sequences from GenBank along with the sequences originated from this work. The Hasegawa–Kishino–Yano model was applied, and maximum likelihood (ML) bootstrap values with 1000 replicates were estimated for statistical robustness. This model was determined to be the most effective replacement model by Mega X [23].

### 2.6. Statistical Analysis

The occurrence of *E. bieneusi* in animals from the two zoos in Portugal was determined by calculating the proportion of positive samples relative to the total samples analyzed, along with a 95% confidence interval (95% CI). 

## 3. Results

From the analysis of the 127 fecal samples, 1.57% (2/127; 95% CI: 0.19–5.57) were positive for *E. bieneusi*. The two positive samples derived from two NHPs from Maia Zoo: a *Hylobates lar* (white-handed gibbon) and a *Lemur catta* (ring-tailed lemur). Both were the sole representatives of their species in the zoos. The occurrence in NHPs was 18.18% (2/11; 95% CI: 2.28–51.78), while in all other animal species, the occurrence was 0%. 

The *E. bieneusi* sequences derived from the white-handed gibbon and the ring-tailed lemur were deposited in GenBank under accession numbers PP150515 and PP150514, respectively. Pairwise nucleotide sequence similarity of the two positive samples obtained in the present study was 96.33%.

BLAST analysis of the obtained sequence from the white-handed gibbon (PP150515) showed highest match (100% identity) with *E. bieneusi* genotype ERUH4 (MT193680), obtained from a horse in Turkey in 2020. The sequence retrieved from the ring-tailed lemur (PP150514) showed the highest match (97.02% identity) with *E. bieneusi* genotype BEB6 (MK982508), obtained from a calf in Bangladesh in 2020. 

**Figure 1:**
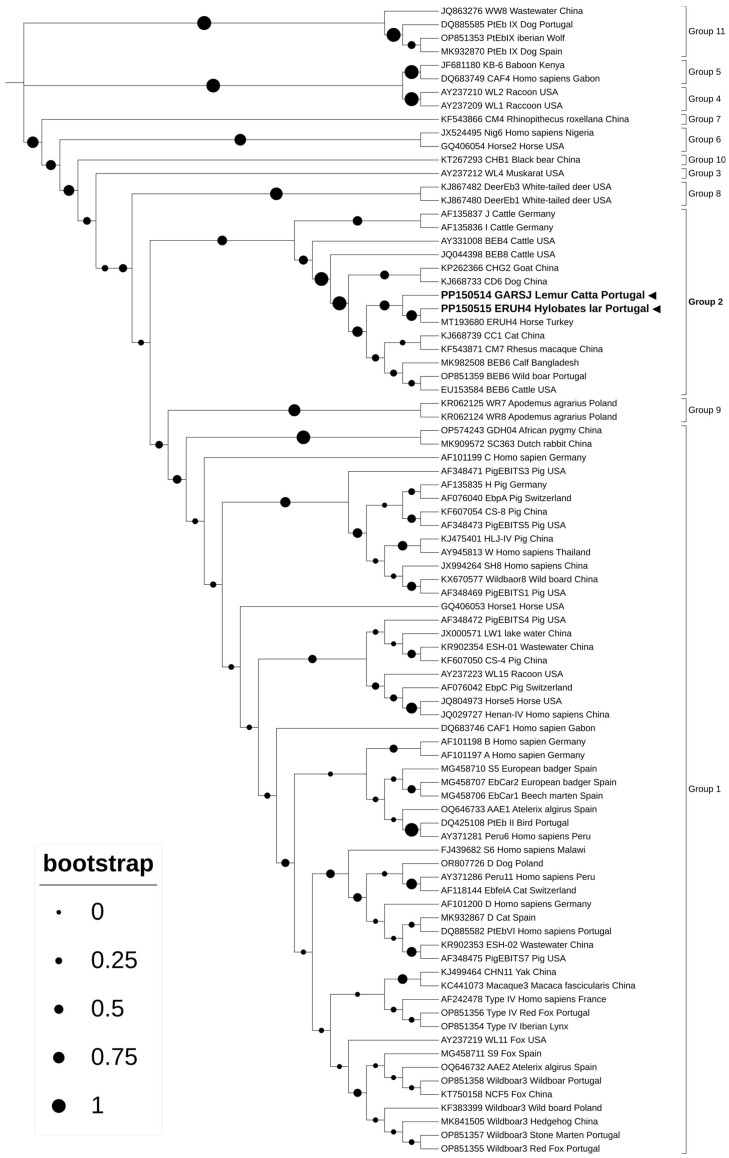
Phylogenetic analysis of *Enterocytozoon bieneusi* sequences obtained in this study (highlighted in bold) and reference genotypes, identified with the respective accession numbers, genotype, host and country of origin. Phylogenetic tree was performed using the maximum likelihood method and the Hasegawa–Kishino–Yano model.

Phylogenetic analysis of the obtained ITS amplicons showed that the sequences originated in our study grouped with sequences from Group 2 (Figure 1). Genotype ERUH4 and a proposed novel genotype denominated here as “GASRJ” were identified from the white handed gibbon and the ring-tailed lemur, respectively

## 4. Discussion

In this study, the occurrence of *E. bieneusi* was assessed in 127 zoo animals from mainland Portugal, with molecular characterization of the detected variants also conducted.

In Portugal, *E. bieneusi* has been detected in several animal species, including domestic, wild and zoo animals, in a total of 13 genotypes identified (BEB6, Peru6, PtEb IV, PtEb V, D, PtEb VII, PtEb VIII, PtEb IX, PtEb X, PtEb XI, PtEb XII, Type IV, and Wildboar3) [25,26,27,28]. To date, only one study in the country has reported *E. bieneusi* in zoo animals in 2006 [26]. In that study, the presence of *E. bieneusi* was confirmed molecularly in fecal samples from a marmoset (*Callithrix geoffroyi*) and a Kudo (*Tragelaphus strepsiceros*), identified as genotypes PtEb XII and PtEb V, respectively. 

In our study, the positive samples derived from NHP’s (white-handed gibbon and ring-tailed lemur) and the overall occurrence of *E. bieneusi* was 1.57% from the 127 fecal samples tested, and 18.18% in NHP’s. Recent molecular studies conducted in China and Kenya revealed the pathogen’s common occurrence and considerable genetic diversity among NHP’s [2,20]. The majority of these genotypes belong to genotypic Group 1, some of which have been detected in humans worldwide, raising concerns about the potential role of NHP’s in the zoonotic transmission of *E. bieneusi*. A previous study conducted in seven zoos in China tested 496 NHP’s fecal samples, using an ITS-based PCR and sequence analyses. From the 36 NHP’s species from nine families tested, *E. bieneusi* was detected in 29.8% of the samples, including in the same species as detected here: 24.4% (11/45) in ring-tailed lemur and 62.5% (5/8) in white-handed gibbon [2]. However, the genotypes identified in the present study differed from those found in the study performed in China. Specifically, genotypes Type IV, EbpA, O, CM16, CM10, CM11, and CM18 were found in the ring-tailed lemur, whereas genotypes EbpC, EbpA, BEB4, and CM17 were found in the white-handed gibbon [2]. Other studies have also found evidence of *E. bieneusi* in NHP’s. In a study from China, 12.5% of the 369 fecal samples from NHP’s tested positive for *E. bieneusi*, specifically from rhesus macaques and northern white-cheeked gibbons [29]. A study on captive and semi-captive NHP’s in Côte d’Ivoire, Sierra Leone, and Peru, in a total of 116 specimens, only detected one positive animal, a sooty mangabey, from Côte d’Ivoire (4.2%; 1/24) [30]. An investigation carried out in six European zoological gardens from France, Germany, and Spain, involving 35 genera of NHP’s (*n* = 454) as well as their zookeepers (*n* = 70), detected an occurrence of *E. bieneusi* of 0.9% in NHP’s: two gorillas, a saguinus and a saimiri [31]. The occurrences observed in the different mentioned studies, including ours, show occurrences ranging from 0.9% to 29.8%. It is noteworthy that caution should be taken when analyzing the results obtained in this study, given the small sample size of NHP’s. Moreover, in the present study, only feces with a well-formed structure and no other signs of gastrointestinal disease were collected which may have reduced the number of detected *E. bieneusi*. Lastly, genomic DNA isolation from feces is known to be difficult, possible inhibitors in feces may affect the results of the study and produce false negatives.

BLAST analysis of the sequence derived from the white-handed gibbon showed 100% identity with *E. bieneusi* sequence genotype ERUH4 from a horse from Turkey (MT193680). The analysis of the entire ITS region allowed the confirmation of the sequence from the white-handed gibbon as genotype ERUH4. This sequence differs in a single SNP (single nucleotide polymorphism) with genotype BEB6. On the other hand, the sequence retrieved from the ring-tailed lemur showed highest identity (97.02%) with sequences of *E. bieneusi* genotype BEB6 from a calf from Bangladesh (MK982508), and a tan sheep from China (MK322762). Further analysis of the ITS region supported the existence of a new genotype, named “GASRJ”. Despite the white-handed gibbon and the ring-tailed lemur belonging to the same zoo, they do not share enclosures. The fact that positive animals do not share the same enclosure, and the sequences exhibit a 96.33% pairwise nucleotide distance, may suggest that the origin of the positive samples is distinct.

The phylogenetic analysis of the ITS amplicons obtained in this study revealed that the sequences clustered with the group containing potentially zoonotic genotypes of *E. bieneusi*, Group 2. Initially considered ruminant-adapted, Group 2 genotypes have since been found in humans and various other animals, including NHP’s [10,19]. Group 1 is the largest group, encompassing genotypes found in both humans and animals. Genotypes within Groups 3 to 13 appear to be more host-adapted and exhibit limited zoonotic potential [19].

The results of the present study enhance our understanding of *E. bieneusi* epidemiology among captive animals in Portugal. The detection of genotypes belonging to the potential zoonotic Group 2 in NHP’s from a zoo underscores their potential role in transmitting this microsporidian to other zoo animals and humans, including zookeepers and visitors. Further studies are required to comprehensively assess the epidemiology of *E. bieneusi* and its impacts, particularly in zoo settings. This is crucial not only due to its potential zoonotic implications but also for the veterinary health of the inhabitants of the zoo, especially considering that the pathological features are poorly understood.

## 5. Conclusions

In conclusion, animals from 39 different families, including birds, mammals, reptiles, amphibians, and arthropods, from two zoological establishments were tested for *E. bieneusi*, with only NHP’s testing positive. The identification of *E. bieneusi* genotypes from Group 2 in NHP’s highlights the necessity for additional research to evaluate the zoonotic potential of the identified genotypes. Regarding zoos, it is important to investigate potential transmission routes and implement strategies for disease prevention and control, including appropriate handling and management practices.

## Figures and Tables

**Table 1:** Oligonucleotides used for the molecular detection of *Enterocytozoon bieneusi*.

Target	Locus	Primer	Sequence (5′-3′)	Reference
*Enterocytozoon bieneusi*	ITS (and flanking rRNA)	EBITS3	GGTCATAGGGATGAAGAG	[22]
		EBITS4	TTCGAGTTCTTTCGCGCTC	
		EBITS1	GCTCTGAATATCTATGGCT	
		EBITS2.4	ATCGCCGACGGATCCAAGTG	

## Data Availability

The data presented in this study are available on request from the corresponding author.

## Supplementary Materials

The following supporting information can be downloaded at: https://www.mdpi.com/article/10.3390/ani14131874/s1, Table S1: Distribution of animal species used in this study.
