# Supplementary material for: Detection of Enterocytozoon bieneusi in Non-Human Primates in Portuguese Zoos
Source: Animals (Basel). 2024 Jun 25;14(13):1874. doi: 10.3390/ani14131874 (PMC11240438; doi:10.3390/ani14131874)
Supplement: Supplementary file 1 [file animals-14-01874-s001.zip › animals-3050031-supplementary.pdf]

Supplementary Materials:

Table S1. Distribution of animal species used in this study.

Class	Family	Common name (Scientific name)	Nr samples (n)	Origin
Amphibia	Ambystomatidae	Axolotl (*Ambystoma mexicanum*)	3	Maia Zoo
	Hylidae	Red-eyed tree frog (*Agalychnis callidryas*)	1	Maia Zoo
Aves	Anatidae	Mallard duck (*Anas platyrhyncus*)	2	Pedagogical Farm of Canelas
			1	Maia Zoo
		Greylag goose (*Anser anser*)	1	Pedagogical Farm of Canelas
		Egyptian goose (*Alopochen aegyptiaca*)	1	Maia Zoo
		Mandarin duck (*Aix galericulata*)	1	Maia Zoo
	Casuariidae	Emu (*Dromaius novaehollandiae*)	1	Maia Zoo
	Cracidae	Great curassow (*Crax rubra*)	1	Maia Zoo
		Bare-faced curassow (*Crax fasciolata Spix*)	1	Maia Zoo
	Estrildidae	Gouldian finch (*Chloebia gouldiae*)	1	Maia Zoo
	Gruidae	Grey crowned crane (*Balearica regulorum*)	1	Maia Zoo
		Red-crowned crane (*Grus japonensis*)	1	Maia Zoo
	Phasianidae	Indian peafowl (*Pavo cristatus*)	1	Maia Zoo
		Chicken (*Gallus gallus*)	4	Pedagogical Farm of Canelas
		Wild turkey (*Meleagris gallopavo*)	1	Pedagogical Farm of Canelas
		Golden pheasant (*Chrysolophus pictus*)	1	Pedagogical Farm of Canelas
	Phoenicopteridae	Lesser flamingo (*Phoenicopterus minor*)	1	Maia Zoo
	Psittacidae	Blue-and-yellow macaw (*Ara ararauna*)	1	Maia Zoo
		African grey parrot (*Psittacus erithacus erithacus*)	1	Maia Zoo
		Burrowing parrot (*Cyanoliseus patagonus*)	1	Maia Zoo
		Rainbow lorikeet (*Trichoglossus haematodus*)	1	Maia Zoo
	Psittaculidae	Grey parrot (*Psittacus grandis*)	1	Maia Zoo
	Rheidae	Greater rhea (*Rhea americana*)	1	Maia Zoo
Diplopoda	Spirostreptidae	Giant centipede (*Archispirostreptus gigas*)	1	Pedagogical Farm of Canelas
Mammalia	Bovidae	Domestic goat (*Capra hicus*)	6	Pedagogical Farm of Canelas
		Domestic goat (*Capra hicus*)	1	Maia Zoo
		Domestic sheep (*Ovis aries*)	5	Pedagogical Farm of Canelas
	Callithrichidae	Common marmoset (*Callithrix jacchus*)	3	Maia Zoo

	Camelidae	Alpaca (*Vicugna pacos*)	1	Maia Zoo
	Cebidae	Capuchin monkey (*Cebus capucinus*)	1	Maia Zoo
	Cercopithecidae	Vervet monkey (*Chlorocebus pygerythrus*)	1	Maia Zoo
		Red-tailed monkey (*Cercopithecus ascanius*)	1	Maia Zoo
	Cervidae	Muntjac (*Muntiacus muntjak*)	1	Maia Zoo
	Didelphidae	Virginia opossum (*Didelphis marsupialis*)	1	Maia Zoo
	Equidae	Horse (*Equus caballus*)	24	Pedagogical Farm of Canelas
		Donkey (*Equus asinus*)	1	Pedagogical Farm of Canelas
		Grevy's zebra (*Equus grevyi*)	1	Maia Zoo
	Felidae	Lion (*Panthera leo*)	2	Maia Zoo
		Tiger (*Panthera tigris*)	3	Maia Zoo
		Eurasian lynx (*Lynx lynx*)	1	Maia Zoo
	Herpestidae	Meerkat (*Suricata suricatta*)	1	Maia Zoo
	Hylobatidae	White-handed gibbon (*Hylobates lar*)	2	Maia Zoo
	Hystricidae	African crested porcupine (*Hystrix africaeaustralis*)	1	Maia Zoo
	Lemuridae	Ring-tailed lemur (*Lemur catta*)	1	Maia Zoo
		Common brown lemur (*Eulemur fulvus*)	1	Maia Zoo
		Ruffed lemur (*Varecia variegata*)	1	Maia Zoo
	Leporidae	Domestic rabbit (*Oryctolagus cuniculus domesticus*)	2	Maia Zoo
	Macropodidae	Red-necked wallaby (*Macropus rufogriseus*)	1	Maia Zoo
	Mustelidae	Oriental small-clawed otter (*Aonyx cinereus*)	2	Maia Zoo
	Suidae	Domestic pig (*Sus domesticus*)	3	Pedagogical Farm of Canelas
	Ursidae	Brown bear (*Ursus arctos*)	1	Maia Zoo
Reptilia	Agamidae	Bearded dragon (*Pogona vitticeps*)	2	Maia Zoo
			2	Pedagogical Farm of Canelas
		Saharan uromastyx (*Uromastix geyri*)	1	Maia Zoo
	Anguidae	European legless lizard (*Ophisaurus apodus*)	1	Maia Zoo
	Boidae	Yellow anaconda (*Eunectes notaeus*)	1	Maia Zoo
	Colubridae	California kingsnake (*Lampropeltis getula californiae*)	2	Maia Zoo
		Milk snake (*Lampropeltis triangulum*)	1	Maia Zoo
		Western rat snake (*Pantherophis obsoletus*)	1	Maia Zoo
		Corn snake (*Pantherophis guttatus*)	1	Maia Zoo
	Eublepharidae	Leopard gecko (*Eublepharis macularis*)	1	Pedagogical Farm of Canelas
	Gerrhosauridae	Giant plated lizard (*Zonosaurus maximus*)	1	Maia Zoo
	Iguanidae	Green iguana (*Iguana Iguana*)	2	Maia Zoo
	Pythonidae	Ball python (*Python regius*)	1	Maia Zoo
		Carpet python (*Morelia spilota variegata*)	2	Maia Zoo

		Burmese python (*Python biviattus*)	3	Maia Zoo
		Reticulated python (*Python reticulatus*)	1	Maia Zoo
	Scincidae	Eastern blue-tongued skink (*Tiliqua scincoides*)	1	Maia Zoo
		Eastern water skink (*Egernia striolata*)	1	Maia Zoo
	Teiidae	Nile monitor (*Salvator marinae*)	1	Maia Zoo
	Testunidae	African spurred tortoise (*Geochelone sulcata*)	1	Maia Zoo
**Total**			127	
